# Variable Temperature Stress in the Nematode *Caenorhabditis elegans* (Maupas) and Its Implications for Sensitivity to an Additional Chemical Stressor

**DOI:** 10.1371/journal.pone.0140277

**Published:** 2016-01-19

**Authors:** Nina Cedergreen, Nils Jakob Nørhave, Claus Svendsen, David J. Spurgeon

**Affiliations:** 1 Department of Plant and Environmental Sciences, University of Copenhagen, Thorvaldsensvej 40, 1871 Frederiksberg C, Copenhagen, Denmark; 2 Centre for Ecology and Hydrology, Maclean Building, Benson Lane, Crowmarsh Gifford, Wallingford, Oxfordshire OX10 8BB, United Kingdom; NIEHS/NIH, UNITED STATES

## Abstract

A wealth of studies has investigated how chemical sensitivity is affected by temperature, however, almost always under different constant rather than more realistic fluctuating regimes. Here we compared how the nematode *Caenorhabditis elegans* responds to copper at constant temperatures (8–24°C) and under fluctuation conditions of low (±4°C) and high (±8°C) amplitude (averages of 12, 16, 20°C and 16°C respectively). The DEBkiss model was used to interpret effects on energy budgets. Increasing constant temperature from 12–24°C reduced time to first egg, life-span and population growth rates consistent with temperature driven metabolic rate change. Responses at 8°C did not, however, accord with this pattern (including a deviation from the Temperature Size Rule), identifying a cold stress effect. High amplitude variation and low amplitude variation around a mean temperature of 12°C impacted reproduction and body size compared to nematodes kept at the matching average constant temperatures. Copper exposure affected reproduction, body size and life-span and consequently population growth. Sensitivity to copper (EC_50_ values), was similar at intermediate temperatures (12, 16, 20°C) and higher at 24°C and especially the innately stressful 8°C condition. Temperature variation did not increase copper sensitivity. Indeed under variable conditions including time at the stressful 8°C condition, sensitivity was reduced. DEBkiss identified increased maintenance costs and increased assimilation as possible mechanisms for cold and higher copper concentration effects. Model analysis of combined variable temperature effects, however, demonstrated no additional joint stressor response. Hence, concerns that exposure to temperature fluctuations may sensitise species to co-stressor effects seem unfounded in this case.

## Introduction

Organisms living in natural environments are regularly exposed to sub-optimal conditions leading to physiological stress that can affect life history parameters, population growth rates and ultimately carrying capacities [1,2]. Studies to describe and understand the mechanisms that link environmental stress to organism biology have to date largely focussed on single continuous stressors. While such simple scenarios may be amenable to generating mechanistic understanding, such cases rarely reflect the field situation [3]. Under the variable stressor conditions that may dominate in the field, the ability of any species to survive may be determined not only by the average conditions that prevail, but also by the amplitude and frequency of change. Indeed, variable environmental conditions may in themselves be stressful. For example, remodelling of membrane lipid composition to maintain appropriate fluidity at specific temperatures [4,5,6], or adaptation of enzyme and transporter compositions to cope with specific chemical conditions [7], can be physiologically costly under rapidly fluctuation. This can result in additional costs for adaptation compared to organisms living under more stable conditions. Such effects may affect physiology directly, or may compromise the ability of organisms to cope with the presence of other stressors in multiple stressor scenarios [8,9,10].

Temperatures can vary geographically and maximum and minimum temperature ranges can fluctuate diurnally by more than 20°C and annually by up to 50°C at any given location. Hence, for resident species, exposure to variable and potentially stressful temperatures is likely to be a reality at least some of the time. The effect of these variations on species may be both direct and indirect; the latter, for example, by changing the way that species cope with a second stressor. To date studies concerning variable temperatures and other stressors have often focussed on interactions with pathogens [11]. Few have so far looked at how variable temperature changes the consequences of chemical exposure. Indeed while many studies have looked at how constant temperature affects toxicity, most frequently noting increasing severity of effect with increasing temperature [3,12,13,14], comparisons of toxicity under constant and variable temperatures are lacking. In a few cases, results from laboratory toxicity tests conducted under constant conditions have been compared to observed toxicity in field studies [15,16]. However in such studies, temperature is one of a range of variable factors considered, and any observed differences cannot be attributed to this single variable.

If there is a physiological cost of optimising biochemistry to changing temperatures, then both a direct effect of variable temperature on a trait response, and also a changed response following exposure to a second stressor may be expected. Here we report the results of a series of toxicity tests for copper with the nematode *Caenorhabditis elegans* conducted at different constant temperatures in the range 8 to 24°C and for variable temperatures on two amplitudes; ± 4°C around mean 12, 16 and 20°C and ± 8°C around mean 16°C. *C*. *elegans* is particularly suited to study work because it has a short-life cycle allowing easy measurement of multiple traits. This biology involves parthenogenetic, temperature independent, productions of a total broodsize of 200–300 eggs during its lifespan, with this maximum number widely attributed to sperm limitation at least under laboratory conditions [17]. Laid eggs hatch to produce larvae that progress through four stages before reproductive age is reached. After reproduction has ended, nematode keeps may live for several days. Hence traits that can be measured included growth, reproduction (brood size) and survival to jointly yield an estimate of population growth rate (PGR). Further, a version of the dynamic energy budget model (DEBkiss) can be used to provide mechanistic understanding of energy fluxes governing changes in growth and reproduction under these different conditions [18]. Four hypotheses could be specifically tested through this approach from our analyses. First, if none of the constant temperature regimes are in themselves stressful, then the observed differences in life history at different temperatures will be simply due to temperature induced changes in chemical rates, described by the Arrhenius equation. To test this hypothesis DEBkiss was used and a temperature factor was applied to the energy fluxes [18]. Second, if a significant proportion of energy is spent adjusting to variable temperature regimes, we expect a decrease in growth and reproduction under fluctuating temperature that will be greater at the large temperature variation amplitude. Third, we may expect Cu toxicity to increase at higher temperatures. This has been shown for nematodes in a previous study (Nørhave et al, 2014), and was proposed to be due either to a higher Cu uptake at high concentrations or to a more severe effect of the Reactive Oxygen Species (ROS) catalysed by Cu. In DEBkiss this mechanisms may be linked to either a reduction in energy acquisition or an increase in maintenance costs. Fourth, interactions between temperature, both constant and variable, and chemical stress follow the model of Independent Action as described by Holmstrup et al. [3]. In DEBkiss this independence will be characterised by model optimal predictions, based on model parameters derived from DEBkiss fits for individual treatments alone.

## Materials and Methods

### Culture conditions for test organism

*C*. *elegans* (N2 Bristol strain) initially from the *C*. *elegans* Genetics Centre, University of Minnesota) were cultivated in darkness at 20°C on nematode growth medium (NGM) agar plates at all time with an excess of *Escherichia coli* of uracil deficient strain OP50 [19]. At least 2 weeks before the start of all experiments new cultures were initiated at the selected test temperatures to acclimatize the worms through several generations. Cultures were maintained by transferring a chunk of agar and associated eggs, juvenile and adult worms from an existing culture to freshly prepared NGM agar plates weekly.

### Set-up of toxicity tests

**Fig 1:**
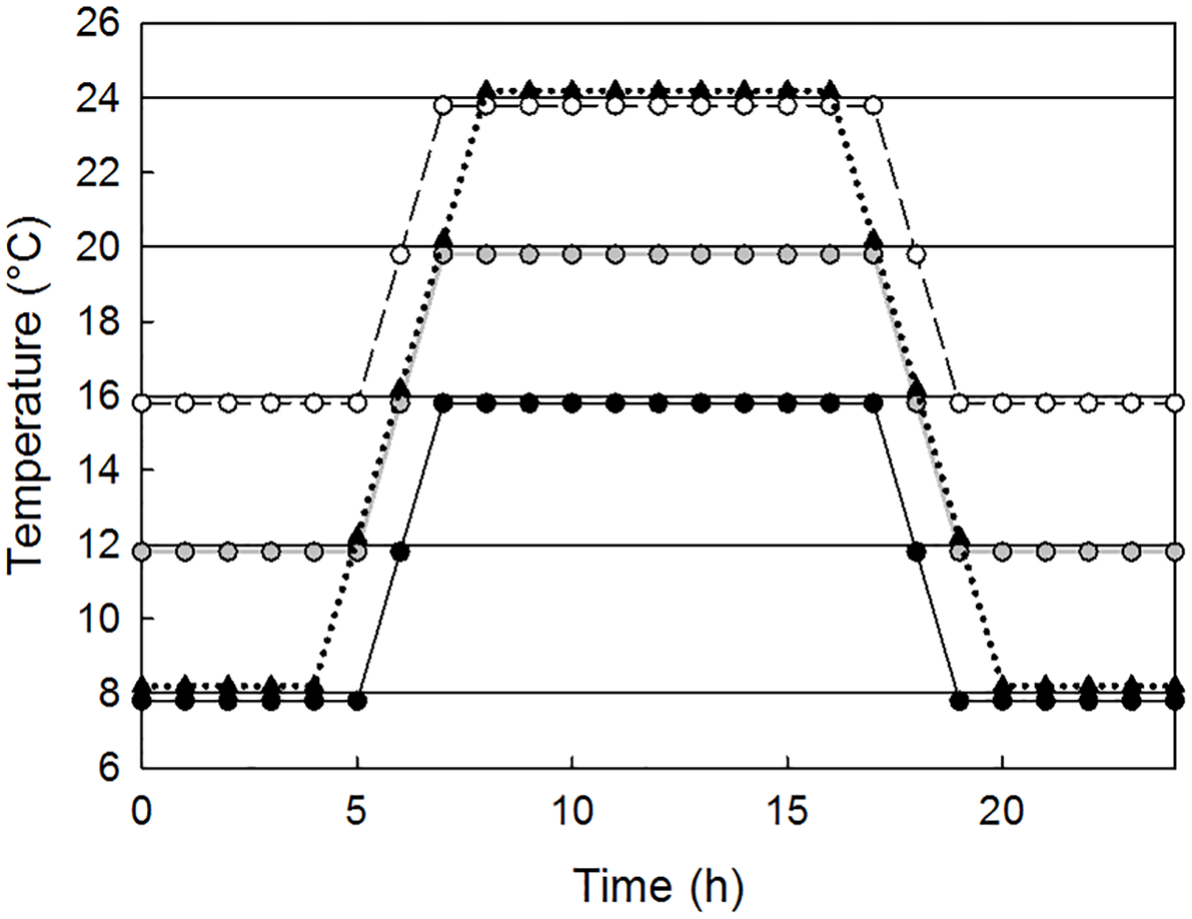
The temperature treatments depicted as temperature as a function of time over 24h. Five constant temperatures (8, 12, 16, 18 and 20°C, solid lines), three temperature ranges with an amplitude of ± 4° around average temperatures of 12°C (black circles, black line), 16°C (grey circle, grey line) and 20°C (white circle, broken line) and one with an amplitude of ± 8°C from the average (black triangles, dotted line) were used. Temperatures were shifted with 4°C per hour. All treatments were run with the minimum and maximum temperature around 12 a.m. and 12 p.m., respectively.

The temperature range selected for study were relevant to the ecological range of *C*. *elegans* [20] and physiological tolerance limits relating to stress on growth and reproductive outputs [21]. Specifically exposures were conducted at steady temperatures of 8, 12, 16, 20 and 24°C and variable temperature scenarios with daily fluctuations of ± 4°C in the ranges 8–16°C (12°C average), 12–20°C (16°C average) and 16–24°C (20°C average) and daily fluctuations of ± 8°C in the ranges 8–24°C (16°C average). Temperatures were changed with a rate of 4°C per hour to simulate changes at two points within a 24 hr period separating two stable temperature periods to provide an simulation of a daily temperature regime under condition of high diurnal variation (Fig 1). Separate toxicity tests with copper were conducted under all temperature regimes using concentration known to result in changes in measured traits [22]. Copper concentrations used were 0, 1, 3, 8, 20 and 40 mg Cu/L agar. The highest concentrations equate to levels 2–3 orders of magnitude greater than those found in surface waters and soil pore waters from uncontaminated sites, but are comparable to soil pore water concentrations in heavily polluted soils.

A stock solution of CuCl_2_ in demineralized water (2 g Cu/L) was made and used throughout the experiment. This copper stock solution was added to the NGM agar while liquid and mixed. Batches of NGM were produced every five days during the course of the experiment to minimize immobilization of copper in the agar, as shown for cadmium by Álvarez *et al*. [23]. All tests were conducted using synchronized cohorts of nematodes produced from adults selected from the main stock cultures acclimatized to each temperature scenario. To generate these cohorts, adult hermaphrodite worms were selected from the acclimatized cultures and placed on Petri dishes with copper spiked NGM and *E*. *coli* and left at the test temperatures for 4 hours (6 hours for 8°C to ensure sufficient eggs were laid). After this time, the adults were removed and the eggs laid in the 4 (6) hour period left to hatch. The offspring (the test organisms), once hatched and grown to the L4 larval stage on the copper spiked plates, were transferred to 12-well plates with copper spiked NGM and *E*. *coli*, with one individual per well. During the test the presence of an *E*. *coli* lawn was always observed at all treatment levels, indicating the provision of food in excess. There were 12 replicate worms for each treatment, except the controls of 24°C constant, 8–16°C, 8–24°C and 16–24°C which had 36 replicates, and the 8°C test in which we could not always obtain 12 worms for each treatment, hence, between 6 and 12 worms were used.

During the reproduction phase of the test, the nematodes were moved to new wells in fresh plates every day. These transfers and the associated observations allowed calculation of the time taken until first egg was laid and subsequently daily egg production. The daily transfer and counting were repeated through both the reproductive and senescence stages until death (determined by the lack of response to being probed). Fertile eggs and hatched juveniles were counted as offspring, while visibly infertile eggs were excluded from brood size counts. At regular intervals, nematode length was measured using a Nikon DS-Fi1 camera connected to a Nikon SMZ 800 stereomicroscope. Body length was measured using the program Nikon NIS Elements Imaging Software 3.2 to provide a description of growth pattern.

### Copper analysis

The copper content in the water fraction of the agar was determined one and five days after production. This allowed us to distinguish dissolved copper and agar bound copper (which may not have been fully bioavailable). A study on *C*. *elegans* focussing on cadmium exposure, has investigated routes of uptake (water versus bacterial food)[24]. This worl concluded that toxic effect correlates best with the Cd concentration in the aqueous phase. As Cu was dosed into the agar as an aqueous solution, we assumed that aqueous Cu concentrations would also be the best measure of exposure in the present study and, hence, analysed this fraction from the agar. Samples of NGM from each produced batch were saved in centrifuge tubes and, after one or five days, centrifuged at 10000 G for five minutes to separate water and agar. A sample of the water fraction was removed and acidified. The analyses were performed on a graphite furnace AAS (Perkin Elmer Zeeman 5100, Waltham, MA) as described in Cedergreen et al, 2013. Blanks, spiked reference samples and a standard reference material NIST 1577c (bovine liver) were included in the analysis to confirm validity [13]. Internal Cu concentrations of nematodes could not be measured, as their small biomass prevented reliable detection.

### Statistics

Differences between temperature treatments for brood size, lifespan and maximal length of non-Cu exposed nematodes were tested by a one-way ANOVA with a Tukey *post hoc* analysis using the R statistical software version 2.12.0 (http://www.r-project.org/).

The average cumulative egg production as a function of time for each treatment was described by a three parameter log-logistic sigmoid model (Eq 1) using the statistical software R version 2.12.0 within the DRC package [25].

y = d/(1+(x/e)b) (1)

where *d* is the maximum number of eggs produced, *e* is the time when half the maximum number of eggs is produced, *b* is proportional to the slope of the sigmoid curve at *e*, *y* is the accumulated number of produced eggs and *x* is the time. From this model the estimated time to first egg (TFE) was calculated by solving *x* for *y* equal to 1/*d*. The DRC package returns a standard error with the estimate.

A stage based matrix population model was constructed for each treatment, based on the daily reproduction values and lifespan. The modelling was performed with the statistical software R version 2.12.0 (http://www.r-project.org/), using the popbio package [26], which is based on the work of Caswell [27]. The models were day staged, thus, making full use of the available reproduction data. A projection of the population over 500 iterations (days) were made and after checking that the population growth had stabilized, the population growth rates (PGR) were determined, with the 95% confidence intervals (CIs) determined by bootstrapping.

To define the effect of Cu on brood size, lifespan, maximum length and PGR, these responses were described with a log-logistic three parameter concentration response model (Eq 1). To assess the joint effect of a potential decrease in any of these parameter as a result of variable temperature and the effect of Cu, the model of independent action was used [28]. The model predicts that if one stressor reduces the endpoint in question to fraction of the unstressed control (R_s1_), and the second stressor affects the endpoint to another fraction of the control (R_s2_), then the unaffected fraction left (R_mix_) when affected by both stressors, is the product of the two unaffected fractions as:

$${\text{R}}_{\text{mix}}{\text{ = R}}_{\text{s1}}{\text{R}}_{\text{s2}}$$ (2)

### The DEBkiss Model and stress interactions

The structure of the DEBkiss model is described in detail in Jager et al [18], with model application to address the effects stressor combinations in *C*. *elegans* (cadmium and fluoranthene toxicity) further elaborated by Jager et al. [29]. The model equations are described in Table 1 and the model parameters in Table 2. Basically, food is assimilated by the maximal rate of J_A_, which scales with the surface area of the organism at a particular given temperature [30]. The assimilated energy is divided in a proportion allocated to maintenance (J_M_) and growth (J_V_), jointly κ, while the remaining energy (1- κ) goes into reproduction (J_R_) when the organism has reached its length of puberty (L_p_) (S1 Fig). Both the rate of assimilation (J_A_) and maintenance (J_M_), and indirectly the rate of growth, as J_V_ = κJ_A_-J_M_, and reproduction as J_V_ = (1-κ)J_A_, will be affected by temperature through the general effect of temperature on chemical reactions governed by the Arrhenius equation. The Arrhenius equation states that the chemical rate *k* can be described by:

$$k=Ae^{-(Ea/RT)}$$ (3)

Where *A* is a pre-factor, *E*_*a*_ is the activation energy of a chemical reaction, *R* is the gas constant and *T* is the absolute temperature given in °K [31]. If the energy fluxes of the DEBkiss model are determined at a standard temperature, as for example 20°C (293.15°K), then the change of the rate of the fluxes (J_A_ and J_M_) with temperature, can be estimated by multiplying them with a temperature factor. The factor (*F*_*T*_), given by the difference between the rate at the reference temperature *k*_*ref*_ and the ambient temperature *k*_*a*_, is given by:

$$k_{ref}-k_{a}=e^{\left(\frac{Ea(1/R)}{T_{Ref}}-\frac{Ea(1/R)}{T_{a}}\right)}=F_{T}$$ (4)

*T*_*ref*_ is the reference temperature given in °K and *T*_*a*_ is the ambient temperature given in °K. *E*_*a*_*(1/R)* is a constant named the Arrhenius temperature (T_A_) in a DEB-context, and typically lies within 5000 and 13,000 K [32]. To describe the data from the five constant temperatures, first robust parameter estimates were obtained on the 20°C stable temperature treatment without Cu. As the experiments were run in parallel with those published by Jager et al (2014), and the 20°C control treatments were overlapping, the parameters estimated by Jager et al (2014) were used as starting values. Contrary to Jager et al (2014) we did not include an initial food limitation, as we did not have length measurements at the initial growth stages to support parametrisation. If, however, we assumed Van Bertalanffy growth started one day after hatching, growth data was very well described without the initial food limitation. When robust parameters were obtained for the 20°C control data, the 8, 12, 16 and 24°C data was included, and the temperature factor *F*_*T*_ was multiplied with *J*^*a*^_*Am*_ and *J*^*v*^_*M*_ to determine the approximate size of the Arrhenius temperature (*T*_*A*_). Finally, all parameters were optimised jointly using a Monte Carlo Markov Chain optimisation procedure.

**Table 1:** Equations for the basic DEBkiss model.

Model component	Specification
Fluxes in mg (dw) d^-1^	
Assimilation	*J*_*A*_ = *fJ*^*a*^_*Am*_ *L*^*2*^
Maintainance	*J*_*M*_ = *J*^*v*^_*M*_ *L*^*3*^
Structral growth	*J*_*v*_ = *y*_*VA*_*(κJ*_*A*_*-J*_*M*_*)*
Reproduction	*J*_*R*_ = *(1-κ)J*_*A*_ *for L>L*_*p*_
State variables in mg (dw)	
Structural body mass	*d/dt W*_*V*_ = *J*_*V*_ with *W*_*V*_(0)*≈* 0
Assimilate buffer in egg	*d/dt W*_*B*_ = *-J*_*A*_ with *W*_*B*_(0) = *W*_*B0*_
Reproduction buffer	*d/dt W*_*R*_ = *J*_*R*_ with *W*_*R*_(0) = 0
Chemical state variable in μg/mL	
Internal chemical concentration	*d/dt C*_*v*_ = *k*_*e*_*(L*_*m*_*/L)(C*_*d*_*-C*_*v*_*)-(C*_*v*_*/W*_*v*_*)d/dtW*_*v*_
Conversions	
Volumetric length to dry weight	*W*_*V*_ = *d*_*V*_ *L*^*3*^
Volumetric length to physical length	*L*_*W*_ = *L/δ*_*M*_
Temperature factor	*F*_*T*_ = *e*^*(TA/Tref—TA/Ta)*^
Maximum size of the control	*L*_*m*_ = *κJ*^*a*^_*Am*_*/J*^*v*^_*M*_
Stress function related to scaled internal Cu^1^	*s*_*Cu*_ = *1/C*_*T*_*max(0*, *C*_*v*_*-C*_*o*_*)*
Applying stress factors to model parameters	
Stress on assimilation flux	*J*^*a*^_*Am*_*-stress = J*^*a*^_Am_*max(0,*1-s)*
Stress on metabolic flux	*J*^*V*^_*M*_*-stress = J*^*V*^_*M*_*(1+s)*
Stress on cost of growth	*y*_*VA*_ *-stress = y*_*VA*_*/(1+s)*
Stress on cost of reproduction	*y*_*BA*_ *-stress = y*_*BA*_*/(1+s)*
Stress on length at puberty	*L*_*p*_*-stress = L*_*p*_*(1+s)*

^1^ The use of scaled concentrations rather than measured is discussed in Jager et al. [33]

**Table 2:** Parameters of the DEBkiss model as used in this study. The fixed parameters were retrieved from Jager et al (2014) and were the same for all parameter fits. The fitted parameters are given for the three scenarios: 1) All constant temperature data (minus 8°C) are described jointly using the Arrhenius temperature and 20°C as a reference, 2) The constant 16°C and the variable 16±8°C treatments are described together applying a stress factor (*s*) on the somatic maintenance rate and 3) The 16°C treatment is described in the presence of the three highest Cu concentrations applying a Cu-related stress factor (*s*_*Cu*_, Table 1) on maximum assimilation rate (*J*^*a*^_*Am*_) and length at puberty (*L*_*p*_). Parameter estimates are given ± s.d.

Symbol	Description	Constant temp. Ref. 20°C	16°C constant and ±8°C	16°C and Cu	Unit
Conversion factors					
*d*_*V*_	Dry weight density of structure	0.25			mg mm^-3^
*δ*_*M*_	Shape-correction coefficient	0.12			-
Fixed model parameters					
*f*	Scaled functional response	1			-
*L*_*0*_	Length at hatching	0.21			mm
*T*_*ref*_	Reference temperature	293			°K
*T*_*a*_	Ambient temperature	281–297			°K
*y*_*BA*_	Yield of egg buffer on assimilation	0.95			mg mg^-1^
*y*_*VA*_	Yield of structure on assimilates	0.80			mg mg^-1^
*C*_*d*_	External Cu concentration	0–40			mg L^-1^
Parameters fitted to growth and reproduction					
*κ*	Allocation fraction to soma	0.734±0.011	0.751±0.019	0.696±0.017	-
*J*^*a*^_*Am*_	Specific maximum assimilation rate	0.094±0.001	0.109±0.008	0.086±0.002	mg mm^-2^ d^-1^
*J*^*v*^_*M*_	Specific somatic maintenance rate	0.386±0.001	0.531±0.046	0.352±0.015	mg mm^-3^ d^-1^
*L*_*p*_	Physical length at puberty	0.962±0.007	1.057±0.009	1.057*	mm
*T*_*A*_	Arrhenius temperature	9109±80	-	-	K
*W*_*B0*_	Dry weight of a single egg	5.67±0.12	10.8±1.2	7.28±0.23	ng
Stress due to variable temperature					
*s*	Stress factor	-	0.076±0.010	-	-
Toxicological parameters for Cu					
C_0_	No-effect threshold	-	-	<0.001	mg L^-1^
C_T_	Tolerance concentration	-	-	89±6	mg L^-1^
k_e_	Elimination rate constant	-	-	10**	d^-1^

* Fixed at the value of 16°C constant temperature experiment

** The elimination rate constant was approaching unrealistic high values and was therefore fixed to 10, signifying a very rapid time to internal equilibrium in the nematodes.

To test whether the stress induced by the high amplitude fluctuations mainly affected assimilation or the cost of maintainance, growth or reproduction, a general stress parameter, *s*, was applied to either *J*^*a*^_*Am*_, *J*^*v*^_*M*_, *y*_*VA*_ or *y*_*BA*_ as described in Jager and Zimmer [34]. First parameters were optimised for the 16°C constant treatment, and then both constant and the 16 ± 8°C treatment were fitted together including the stress parameter on one of the above variables and optimised jointly using a Monte Carlo Markov Chain optimisation procedure.

The effect of Cu on growth and reproduction was tested on the 16°C constant temperature treatment using the three highest Cu-treatments: 8, 20 and 40 mg Cu L^-1^. The 1 and 3 mg Cu L^-1^ treatments were omitted as they induced an increase in reproduction, which the DEBkiss model cannot account for as it assumes a negative effect of an applied chemical. To describe the effect of Cu, a Cu-related stress parameter, *s*_*Cu*_, was applied first to all the main DEB parameters (*J*^*a*^_*Am*_, *J*^*v*^_*M*_, *y*_*VA*_ or *y*_*BA*_), none of which described both growth and reproduction really well. Then, as it was observed that the size of the nematodes at the time where they laid their first eggs decreased with increasing Cu-concentrations (which is contrary DEB-assumptions of constant size at first reproduction) [34,35], the stress factor was also applied to the parameter: length a puberty (*L*_*p*_) by multiplying it with 1-s_Cu_. To predict the joint effect of both variable temperature and Cu-stress, the Cu-model was run including the stress factor obtained for the variable stress, and the predictions were assess together with the data from the multiple stress experiment. All modelling was done using OpenModel 2.2.0.

## Results

### Chemical analyses

All measured Cu concentrations were within the range of 81–110% of nominal concentrations with an average of 95 ± 5% (mean ± s.d., *n* = 51). This indicates a high level of agreement between nominal and measured concentration and hence hereafter nominal concentrations are used for all calculations and discussions.

### Are the constant temperatures in themselves stressful?

**Fig 2:**
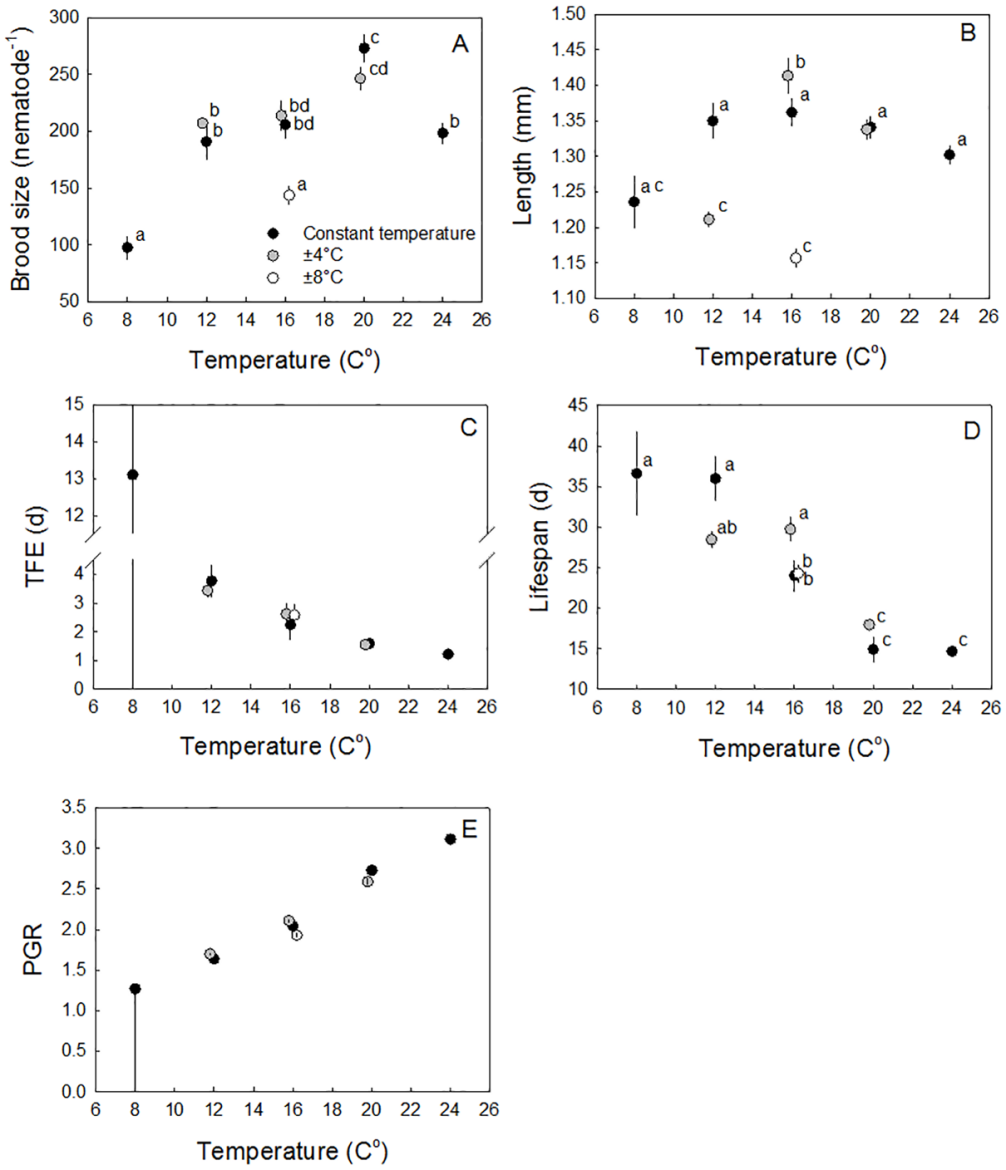
Temperature effect on five different endpoints: final brood size (A), Final body length (B), time to first egg (C), lifespan (D) and Population Growth Rate (PGR) (E). All data are given as a function of the mean temperature of the treatment. Data are given as mean ± s.e.m., apart from PGR which is given with 95% Confidence Intervals (CI) obtained by bootstrapping. Significantly different treatments (ANOVA followed by a Tukey *post hoc* test) are denoted by different letters. Constant temperature treatments are given in black symbols, treatments varying ± 4°C are given in grey symbols and treatments varying ± 8°C are given in open symbols.

**Fig 3:**
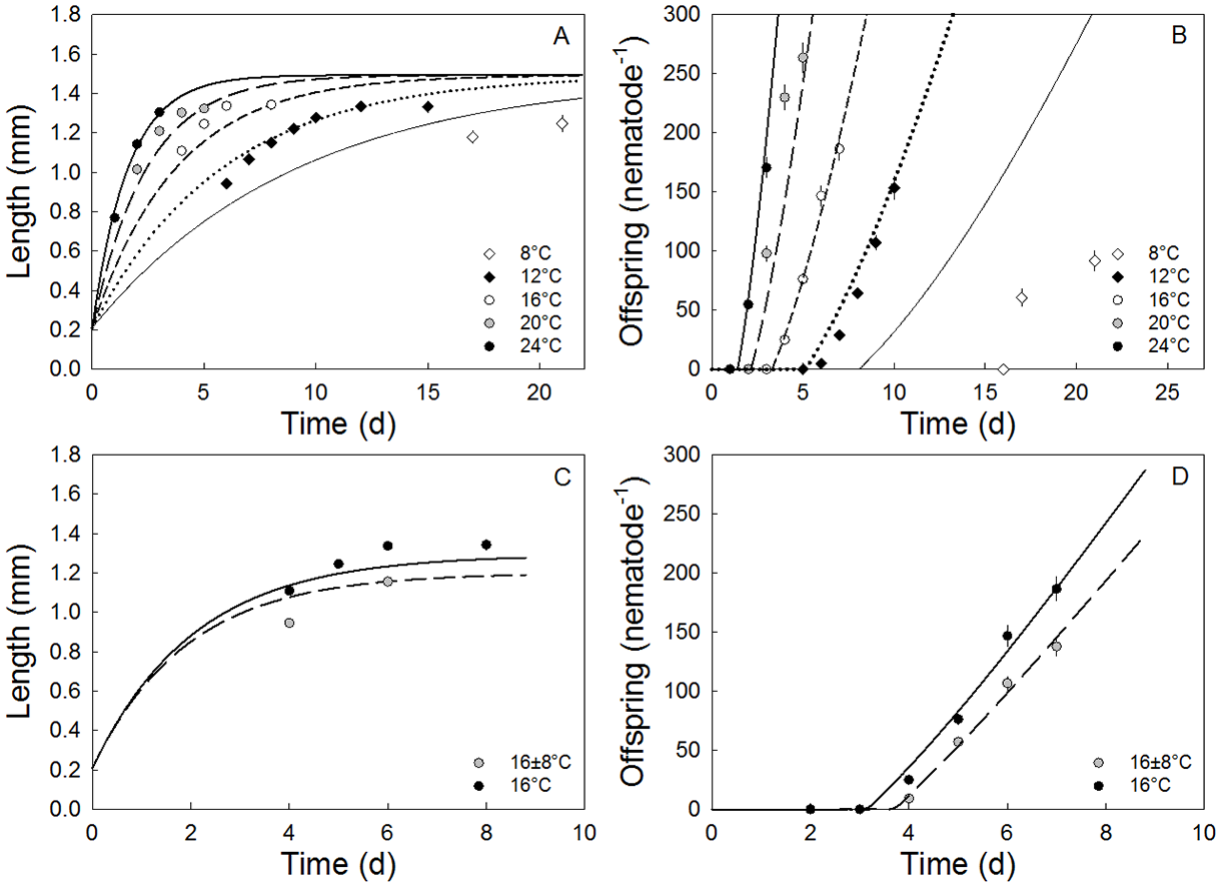
Temperature effects on traits fitted using the DEBkiss model. The development of body length (A) and offspring production (B) as a function of time for the five constant temperature treatments are described by the DEBkiss model including a temperature function (R^2^ = 0.87). Fig C and D shows bodylength and offspring production as a function of time for the constant 16°C treatment and the variable 16 ± 8°C treatment described by the DEBkiss model including an 8% increase in mainainance cost for the variable treatment (R^2^ = 0.87). Data are given as mean ± s.e.m. Model parameters are shown in Table 2.

Brood sizes were similar at 12, 16 and 24°C even though increasing temperature shortened brood period, while nematodes in the 8°C treatment produced a broodsize half those of the any temperature treatment (Fig 2A). This indicates a direct chill stress effect, but no heat stress response in the temperature range considered. The 20°C temperature was ultimately optimal, with nematodes producing on average approximately 20% more offspring than in the 12, 16 and 24°C treatments (Fig 2A). Despite different growth rates, there was only a slight, non-significant, negative relationship between temperature and size at the end of their lifetimes at all constant temperatures in the 12–16°C range. Length were, however, reduced at 8°C contrary to the expectation from the Temperature Size Relationship, further supporting indication of a chill stress (Fig 2B). Both time to first egg (TFE) and lifespan decreased with increasing temperature, making population growth rate (PGR) linearly related with temperature (Fig 2C, 2D and 2E). DEBkiss well described growth and reproduction at 20°C (R^2^ = 0.95). Including the temperature factor, *F*_*T*_, and describing all growth data with the 20°C parameters optimising *T*_*A*_ gave a poorer fit (R^2^ = 0.63), with the 8°C treatment clearly lying below the fitted curves (Fig 3A and 3B). Omitting the 8°C data still using the 20°C parameters increased the R^2^ to 0.83, supporting the indication from the single traits of broodsize and body size that the 8°C was stressful causing deviation from expectation from the Arrhenius relationship alone. All four other constant temperatures accorded well with model predictions (Fig 3A and 3B). All parameters were thereafter optimised for 12, 16, 20 and 24°C constant temperature using a Monte Carlo Markov Chain optimisation (R^2^ = 0.87). The resulting fits and parameter values are given in Fig 3A and 3B and Table 2.

### Variable versus constant temperatures

For the nematodes kept under low amplitude (± 4°C) variable temperatures for the two highest average temperatures of 16 ± 4°C and 20 ± 4°C, changes observed in traits were generally positive in direction, but were not significant between the variable and constant exposure for any endpoints (Tukey: *p* < 0.05, Fig 2A–2D). For the low amplitude (± 4°C) variable at the low temperature range of 12 ± 4°C, both lifespan and maximal length was significantly reduced compared to the constant 12°C temperature treatment (Tukey: *p* < 0.05, Fig 2A and 2D), while TFE and broodsize were not affected (Tukey: *p* > 0.05, Fig 2B and 2C). The high amplitude treatment (± 8°C) at 16 ± 8°C, however, showed a significant reduction in maximal length (15%) and brood size (30%) compared to the 16°C constant temperature treatment. Together with a 15% longer TFE this resulted in a reduction in population growth rates of 5% in the 16 ± 8°C compared to 16°C constant treatment from 2.04 (2.02–2.07) to 1.93 (1.91–1.95)(Fig 2E).

Testing whether an increase in maintenance cost could explain the reduced growth and reproduction using the DEBkiss model, we first fitted the constant 16°C data (R^2^ = 0.95) and then added the dataset from the 16 ± 8°C treatment and a stress factor on the parameter: specific somatic maintenance rate (*J*^*v*^_*M*_). This model described the observed patterns of body size and offspring production in time well (R^2^ = 0.87) if *J*^*v*^_*M*_ was increased by approximately 8% (See parameter values in Table 2, Fig 3C and 3D). Decreasing the maximum assimilation rate (*J*^*a*^_*Am*_) by approximately 6% or increasing the cost of growth given by *y*_*VA*_ by 14%, however, gave equally good fits (R^2^ = 0.87). Hence, while increased maintenance costs could provide an explanation for the observed effects in accordance with our initial hypothesis, this mechanism could not alone be distinguished as the most important process governing the stress effects of the high variable temperature 16 ± 8°C treatment on traits.

### Cu stress at different temperatures

**Fig 4:**
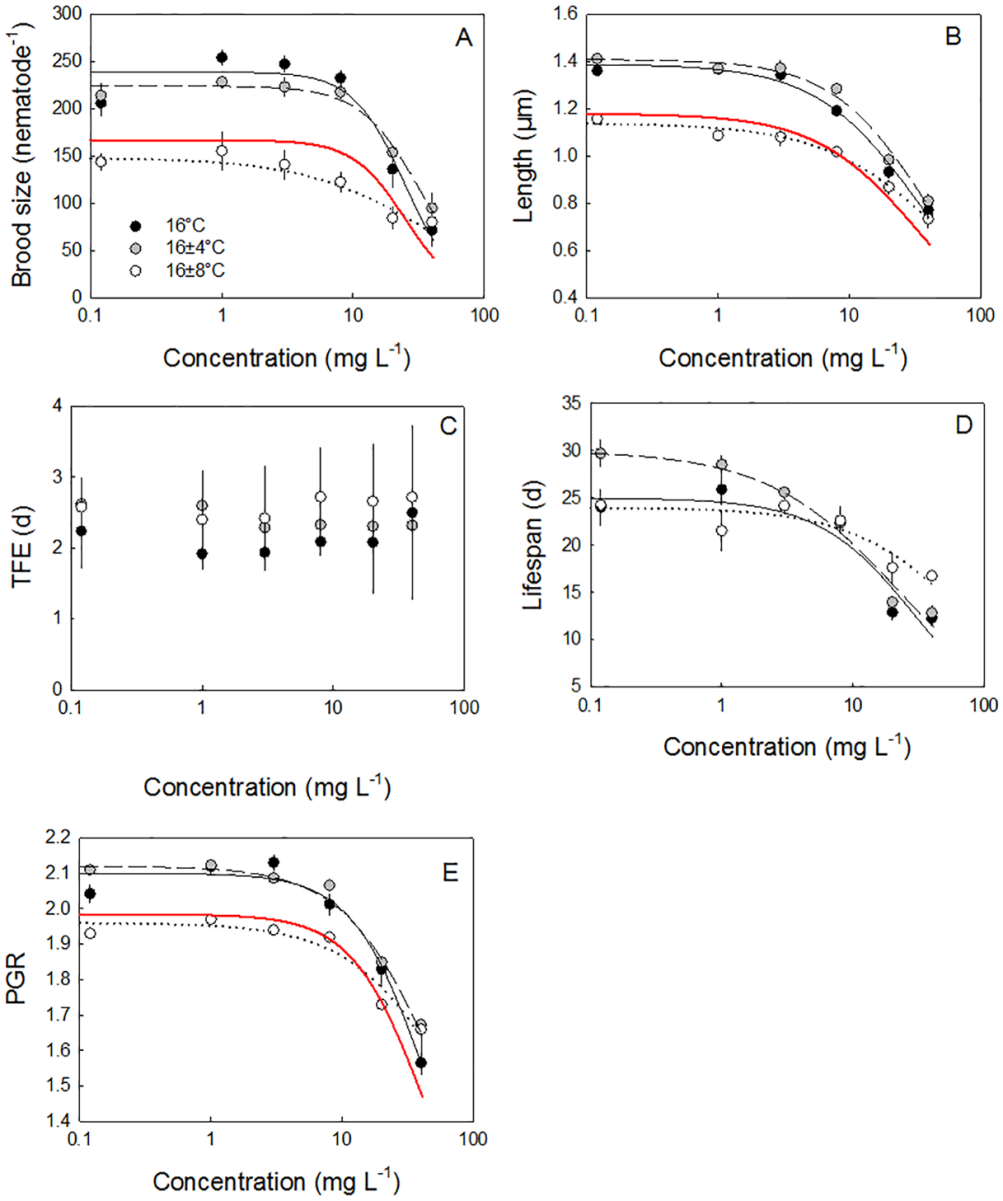
The effects of Cu exposure on traits responses in *C*. *elegans* in treatments with an average temperature of 16°C. The five different endpoints: Final brood size (A), final body length (A), time to first egg (B), lifespan (D) and Population Growth Rate (PGR) (E) for the constant 16°C treatment (filled symbols), the 16 ± 4°C (grey symbols) and the 16 ± 8°C (open symbols) as a function of Cu concentrations in the agar. Data are given as mean ± s.e.m. and are described with a three parameter log-logistic concentration response model, except for TFE. The parameters are given in Table 3, together with the concentration-response parameters of the other temperature treatments.

Copper exposure had a severe effect on growth, reproduction and lifespan mainly at the two highest tested concentrations of 20 and 40 mg Cu L^-1^, while the two low concentrations 1 and 3 mg Cu L^-1^ no effect, or even, in the case of offspring production, a slight increase in trait values was seen (see Fig 4A, 4B and 4D for example data set for the 16°C treatment). No effect on any concentration on TFE was observed (Fig 4C). Integration of all measured effects to assess Cu effect on PGR indicated a reduction also at the two highest tested concentrations (Fig 4E). Reproduction, final body length, lifespan and PGR could be described with concentration-response curves for all studies under the different temperature regimes (Table 3). Consistent with our hypothesis, increasing temperature to constant 24°C resulted in a lower EC_50_ for all responsive traits than at 12, 16 and 20°C, consistent with previous work and expectations [22]. The largest shift in sensitivity was seen for PGR for which the EC_50_ reduced from 61.2 mg Cu L^-1^ at 12°C to 25.5 mg Cu L^-1^ at 20°C. Additional inclusion of a treatment at 8°C, which was not included in the study of Norhave et al. [22], identified an increased Cu sensitivity of nematodes kept at this lower temperature, most notably for broodsize (Table 3). This sensitivity was not consistent with our initial hypothesis of increased sensitivity with increasing temperature.

**Table 3:** Dose-response parameters (Eq 1) for the measured endpoints: Length increase (Maximum length minus length at hatching), lifespan, brood size and population growth rate (PGR) for the different temperature treatments. The curves for the three treatments with an average temperature of 16°C are shown in Fig 3. Data for the 12°C and 24°C average temperature treatments are presented in S2 and S3 Figs. The parameters are given ±SE.

Endpoint	Treatment (°C)	*d*	*b*	*EC*_*50*_
Length increase (mm)				
	8	0.99±0.03	1.45±0.24	25±3
	12	1–14±0.03	1.26±0.21	39±4
	12±4	1.02±0.03	1.18±0.22	45±6
	16	1.18±0.03	1.11±0.16	34±4
	16±4	1.20±0.03	1.21±0.19	38±4
	16±8	0.93±0.03	0.95±0.27	53±11
	20	1.11±0.03	1.17±0.24	46±6
	20±4	1.12±0.03	1.41±0.24	34±3
	24	1.06±0.03	2.56±0.37	23±1
Lifespan (d)				
	8	33±1	12.4±62.2	48±46
	12	36±2	1.29±0.36	35±6
	12±4	30±1	1.35±0.50	54±15
	16	25±2	1.18±0.38	30±8
	16±4	30±1	0.86±0.24	23±6
	16±8	24±2	0.98±0.49	86±66
	20	17±2	0.99±0.49	45±24
	20±4	20±1	1.56±0.65	37±10
	24	16±1	1.74±0.78	22±6
Brood size				
	8	90±21	0.67±0.40	7±7
	12	21±6	3.22±0.63	33±2
	12±4	214±5	1.48±0.22	26±3
	16	23±8	2.18±0.40	25±2
	16±4	224±6	1.87±0.35	33±3
	16±8	148±8	0.93±0.30	35±12
	20	285±6	1.72±0.21	24±2
	20±4	262±7	2.02±0.41	31±3
	24	200±6	3.81±1.60	19±12
PGR				
	8	1.24±0.01	2.98±1.33	26.5±3.4
	12	1.67±0.02	1.59±0.42	61.2±10.6
	12±4	1.71±0.03	1.0±0.43	98.1±51.4
	16	2.10±0.03	1.59±0.33	41.5±4.49
	16±4	2.12±0.02	1.24±0.19	52.1±4.8
	16±8	1.96±0.23	1.10±0.31	74.9±20.1
	20	2.77±0.03	1.39±0.18	44.4±3.4
	20±4	2.70±0.05	1.51±0.37	46.9±6.90
	24	3.19±0.05	3.10±0.49	25.5±1.42

**Fig 5:**
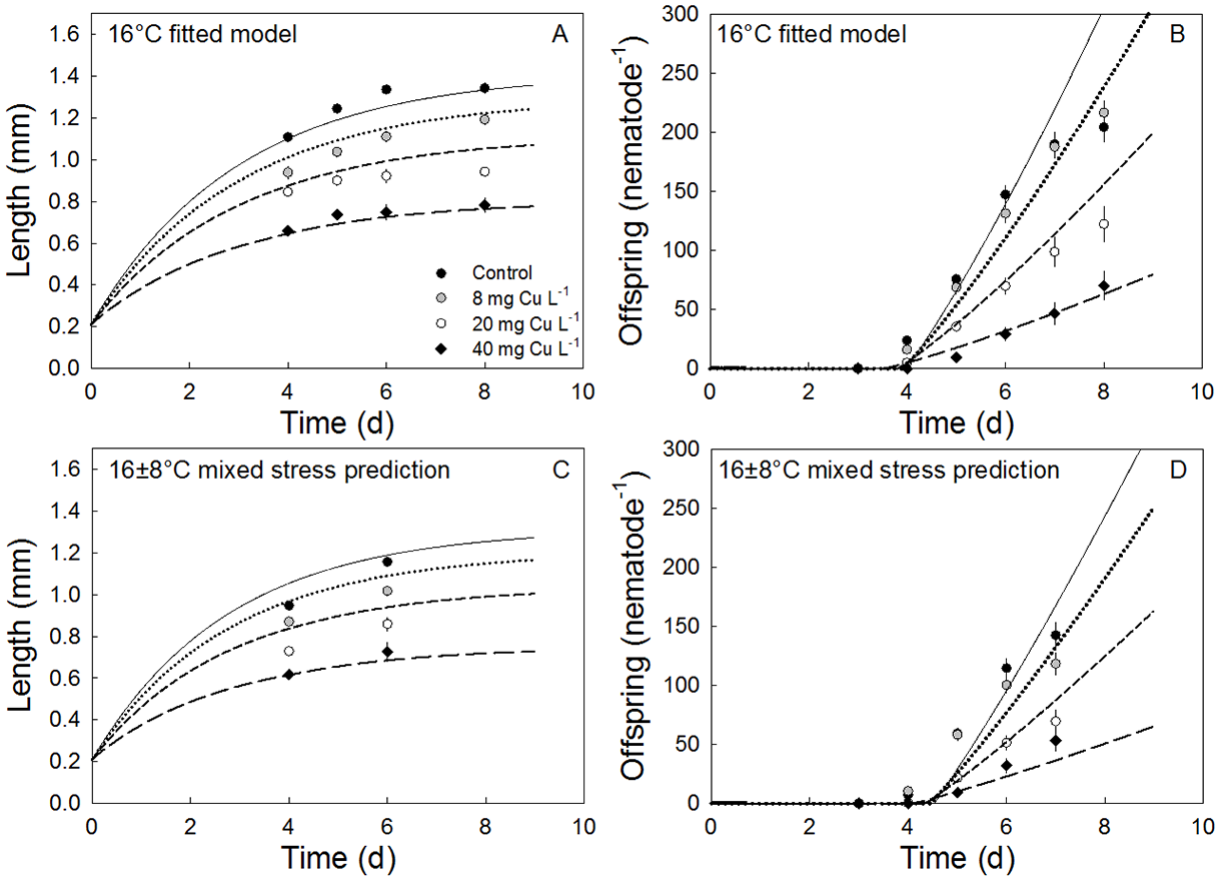
Copper exposure effects on traits fitted using the DEBkiss model. The development of body length (A) and offspring production (B) as a function of time for the 16°C constant treatment combined with 0, 8, 20 and 40 mg Cu L^-1^ described by the DEBkiss model. Fits include a Cu-related stress factor (*s*_*Cu*_, Table 1) on maximum assimilation rate (*J*^*a*^_*Am*_) and length at puberty (*L*_*p*_) (R^2^ = 0.97). A mixed stressor prediction including the 8% increase in somatic maintainance obtained when fitting the constant and variable control treatments together, and the Cu-related stress factor ontained from the constant temperature treatment (A, B) is shown together with the body length (C) and offspring production (D) data from the 16 ± 8°C treatment combined with 0, 8, 20 and 40 mg Cu L^-1^. Jointly the mixed stressor prediction describes 75% of the variation in the data. The 1 and 3 mg Cu L^-1^ data are omitted from the fit, as they increased offspring production, which the DEBkiss model is not parameterised to deal with. Data are presented as mean ± s.e.m.

Describing the effect of Cu using the DEBkiss approach, gave good fits of the responses of body length and offspring production in time by applying a Cu-related stress parameter on on maximum assimilation rate (*J*^*a*^_*Am*_) and length at puberty (*L*_*p*_) (R^2^ = 0.97) for the 16°C treatment (Fig 5A and 5B). Other combinations of stress parameters could potentially also give good fits, but a Cu related stress on energy flowing into the organism is consistent with expectations driven by Cu affects on metabolic systems and ROS affect on physiological processes including the mitochondria [36]. Size at TFE at 16°C constant temperature was observed to decrease from 0.85 mm in non-exposed nematodes to 0.77, 0.70 and 0.60 mm in nematodes exposed to 8, 20 and 40 mg Cu L^-1^ (using the TFE-values for the 16°C constant treatment of Fig 3B and the growth predictions of Fig 5A). These result support identification of a direct impact of copper stress on this trait.

### Interactions between variable temperature regime and Cu stress

To assess how variable temperature regimes affect sensitivity to Cu exposure, we explicitly compared responses for the three treatments with an average temperature of 16°C. Trait responses to Cu exposure in the constant 16°C and low amplitude variation 16 ± 4°C temperatures were remarkably similar for all traits, with only life-span showing difference at the lower exposure levels (longer life-span at low amplitude temperature variation). The high amplitude variable temperature (16 ± 8°C) and the 12 ± 4°C treatment showed lower trait values at 1, 3, 8 and 20 mg Cu L^-1^ treatments, while the 16 ± 4°C and 20 ± 4°C did not show such differences across multiple traits. Further in all treatments, difference in absolute values were not evident for the 40 mg Cu L^-1^ treatment (see Fig 4 for 16 ± 8°C and 16 ± 4°C, S2 Fig for 12 ± 4°C, S3 Fig for 20 ± 4°C). Applying a traditional independent action prediction for the two types of stress: variable temperature and Cu, indicated antagony for the high concentration for length, brood size and PGR (Fig 4). The evaluation of the prediction, however, was limited due to the relative few Cu concentrations producing a significant adverse effect on traits. Nonetheless, the suggested antagonism is further indicated by the EC_50_s for the variable temperature treatments which show higher values than any constant temperature treatment, notably for the 16 ± 8°C and also 12 ± 4°C treatments (Table 3).

The effect of variable temperature on Cu toxicity was further investigated within DEBkiss. Predicting the effect of the mixed stress of variable temperature (± 8°C) and Cu, the increase in maintenance costs obtained from the fit of the constant versus variable 16°C treatment treatments was simply added to the Cu-model to predict the comparative effect of the metal at the constant versus high amplitude variable treatment. The integration of the maintenance effect derived from high amplitude variation with the Cu effect resulted in a model fit that was able to explain 75% of the variation in the data (Fig 5C and 5D). Body length values in the 8 and 20 mg Cu L^-1^ treatment generally fell below the fitted line, while offspring production values at time-points in the 8 mg Cu L^-1^ treatment generally fell above the fitted line. These effects are consistent with the indication of antagonism between stressors that causes deviation of effects from strict independent action.

## Discussion

### Effects of constant temperature regime on toxicity

Constant temperature changed measured *C*. *elegans* life-cycle traits in a manner consistent with expectations from the Arrhenius relationship in the 12°C—24°C temperature range, but not for nematodes at 8°C for which DEBkiss suggested a direct stress effect. For example, maximal length of nematodes shows a slight decrease with increasing temperature in the range 12°C—24°C in a manner consistent with predictions from the Temperature Size Rule. Nematodes growing at 8°C were, however, smaller than individuals grown at higher temperatures indicating of a direct stress effect of cold exposure for this trait that is inconsistent with expectations (Fig 2B). This result questions the generality of the Temperature Size Rule for all ectotherms in a manner consistent with finding from a range of recent studies [37,38]. The linear relationship of PGR with temperature reflects the high sensitivity of this parameter to traits that were strongly positively affected by increased temperature, such at TFE, compared to traits negatively affected by temperature, such as lifespan, to which PGR in *C*. *elegans* is known to be insensitive [39].

The sensitivity to cold at 8°C in a nematode species of such a wide geographical range as *C*. *elegans* [20] observed in results from this study could be related to its native growth environment. Currently characterized populations, such as those known in France [40], demonstrate that *C*. *elegans* prefers nutrient- and microorganism-rich substrates such as rotting fruits and decomposing plant matter [41,42]. These microbial rich environments are the subject of significant warming from heat derived as a by-product of their active microbial communities. Observation of cold, but not heat (up to 24°C) stress are, thus, consistent with expectation based on the known ecology of the species.

That the warmest temperature of 24°C did not in itself prove stressful was itself quite surprising as this temperature is close to temperatures that have previously been shown to affect traits, such as reduced brood size at 25°C [22] and growth, reproduction and survival at 25.5°C [21]. Transition from optimal temperature to stressful temperature at the higher range, therefore, seems to occur within a small temperature range for the species. One practical consequence of our observation is the value of maintaining *C*. *elegans* population at temperatures in the range 12–20°C if the induction of direct temperature induced stress into nematode cohorts used for phenotypic studies is to be avoided. In this respect the implications of the practice of refrigeration storage for phenotypic measurement in later experimental studies may require investigation.

### Effects of variable temperature on traits

Low temperature variations resulted in little change in trait values when compared to worms held under similar average, but constant conditions. This does not support a positive effects on traits related to stress hardening [43], but rather an absence of a variable thermal condition effect on fitness [44]. A direct stress effect of temperature variation on traits was found in two treatments, namely the high amplitude variation 16 ± 8°C and low amplitude variation at the average 12 ± 4°C. The high amplitude variation treatment reduced brood size by 30%, however, despite the magnitude of this effect, PGR was only decreased by 5% (which corresponds to a change of constant temperature from 16°C to 14.2°C based on a regression of PGR as a function of constant temperature, Fig 2E). The relatively low sensitivity of PGR to effects traits such as brood size is consistent with what is known from previous sensitivity analyses of trait relationships to PGR in *C*. *elegans* [23,45].

In was notable that the two variable treatments that showed a direct stress effect included a period where the nematodes were kept at 8°C—a constant treatment that in itself proved stressful. Our hypothesis was that any stress effects of variable temperature would be driven by increased maintenance costs accrued as individuals seek to adapt physiology to the changing temperature conditions. DEBkiss analysis confirmed the possible validity of this hypothesis, however, decreasing assimilation rates or increasing growth costs within DEBkiss could also explain the observed patterns of effect in time with almost a similar level of agreement. Hence, for this case, the DEBkiss model could not unequivocally distinguish between physiological modes of action. Further, and perhaps more fundamentally, that only some variable temperatures (those including time spent at 8°C) affected trait performance point not to a variable temperature effects, but a (maintenance) stress effect just attributable to low temperature exposure

Temperature exposures in poikilothermic organisms are known to have a range of effects on physiology. The obvious relationship is with metabolic rate, which is fundamentally behind the changes observed for some traits (notable TFE and life-span) and PGR as observed in this study. Physiological heat shock can cause the induction of a series of stress response mechanisms, such as the metallothionein, antioxidant defence mechanism and mitogen-activated protein kinase signalling, as well as the heat shock system for protein chaperoning [46]. Cold stress also has a physiological consequence for species. Rapid transfer from warmer to colder temperatures has been shown to stimulate changes in the lipid compliment of species as they attempt to maintain optimal membrane fluidity at different temperatures [4,47]. This remodelling of phospholipid content is naturally subject to time lag during the time when the require lipids are produced in the endoplasmic reticulum and Golgi. Further, during these times of rapid change, membranes may exist for a time in states outside of optimal for the currently experienced temperature conditions. While this has the potential to place organisms under stress, in this case the impacts of these changes on traits actually appear limited. It is only when species have to exist outside their physiologically normal range that stress leading to reduced trait performance results. Variable temperature exposure alone does not appear to be stressful.

### Relationship between Cu toxicity and constant temperature regime

Testing the response of *C*. *elegans* to Cu-stress at the different constant temperatures partly confirmed the study of Nørhave et al [22], who found a 1.5 to 2.5-fold decrease in Cu EC_50_ increasing temperatures from 11 to 24°C, and in addition found stimulations of reproduction and PGA at low Cu concentrations indicative of hormesis. However, going beyond Norhave et al. [22], the inclusion of a lower temperature treatment at 8°C (as compared to the lowest temperature treatment of 11°C used by [22]) showed an increase in sensitivity to Cu at low temperature, based on the EC_50_ values for the different endpoints (Table 3). Hence, an innately stressful temperature regime was shown to increase the sensitivity of *C*. *elegans* to a toxicant. This observation of an interaction between cold stress and chemical exposure has been indicated in a series of studies looking both at how cold exposure affects toxicity and also at how chemical exposure affect cold tolerance [3]. These included specific studies that have looked at these interaction for copper and low temperatures [48,49,50].

DEBkiss modelling of the data for copper exposed nematodes pointed to a number of possible effects on model parameter that could produce patterns of growth and reproduction consistent with observations. Applying a Cu-related stress parameter on maximum assimilation rate (*J*^*a*^_*Am*_) was one such physiological mechanism. Studies of the physiological mode of action of metals in nematodes have pointed to a mechanism that is associated with an effect of the metal on resources acquisition [51,52]. Hence, incorporation of a Cu related stress on energy assimilation is consistent with expectations of metabolic effects on metabolic systems and ROS effects on physiological processes. Copper is recognised for the particular interaction that this metal has with the mitochondria with potential effects on availability of energy through metabolism [36,53].

### Effect of variable temperature regime on Cu toxicity

Our initial hypothesis was that stress related to variable temperature exposure may result in a sensitisation of the nematodes to secondary Cu exposure. However, contrary to expectation, nematodes under the high amplitude variable temperature (16 ± 8°C) actually seem better able to endure exposure to higher Cu concentration than expected by both the independent action and mixed stressor DEBkiss predictions (Figs 4 and 5). Such an antagonistic interaction points to a potential stress hardening effects resulting most likely from exposure to cold. “Stress hardening” effects by which exposure to one stressor may induce mechanisms that protect against the effect of a second stressor have been widely reported for a range of organisms and stressor combinations [9,54,55]. The increased robustness of nematodes exposed to variable conditions including cold stress was also partly supported in the 12 ± 4°C, for which EC_50_-values are higher than those for all other treatments except the high amplitude variable temperature for all traits except brood size (Table 3).

Use of the simple Dynamic Energy Budget model, DEBkiss, allowed a systems-based assessment of the interactions of different stressors with physiologically relevant parameters for both temperature and Cu stress effects. For the exposure to constant and variable temperatures that include a time involving exposure at 8°C, we could not identify one process that explained the decrease in growth and reproduction significantly better than other possible mechanisms. This could be attributed to the low resolution of our data with length monitored only a few points in time, or it could be that the observed temperature effects is driven by some interactions including multiple physiological parameters. Thus, though there are some chemicals and possibly also other stress factors that affect some processes very specifically [51,56], other types of stressors may affect many of the parameters included in DEBkiss simultaneously [57]. Low/variable temperature stress, potentially affecting all enzyme and lipid related processes may be one such general stressor, in contrast to the more specific effect of Cu on assimilation indicated from DEBkiss. We achieved the best combined fit of growth and reproduction by also applying the chemical stress factor to the parameter, length at puberty. This parameter is not one of the traditional parameters for application of stress in a DEB-context [30], where all organisms are expected to start reproducing at a specific size [58]. Both we, and other studies, have shown that organisms ranging from nematodes, daphnids and rotifers decrease their size at first reproduction when exposed to stress as different as starvation, cadmium and copper stress [14,59,60]. Here we do not find the DEB-assumption of constant size at the time of reproduction biologically correct in all cases and, therefore, recommend including size at puberty as one of the parameters possibly affected by stress.

It was relatively easy to combine stressors, making reliable time-dynamic mixed stress predictions using DEBkiss, as also shown in Jager et al. [29]. This identifies DEBkiss as a promising tool for research in combined stressor ecology, particularly those studies involving temperature and, therefore, the rates of physiological processes and their effects on traits over time. Challenges in the implementation of DEBkiss that are recognised include the increased amount of work involved in measuring endpoints continuously over time and the fact that measurements are sometimes in themselves stressful (for example quantifying broodsize in time for nematodes involves regular transfers between newly treated wells). As identified in this study, it may not always be possible to unequivocally identify the physiological changes on energetic parameters that underpin the changes of traits with time. However, this may be a consequence of the underlying biology of the stressor rather than a weakness of the modelling approach.

### Ecological and chemical effect assessment implications

Previous studies that have developed physiological based models to understand the link between changes in resource allocation under (toxicant) stress have provided a set of expected phenotypes for animal subject to different energy allocation related processes and trait effects [61,62,63]. Direct stressor effects on traits could be related to effects on physiological parameters, with low/variable temperature effects driven by the individual stressor and the interaction showing an antagonism. In the field, environmental conditions can be subject to rapid variations that are not included in the laboratory based assessment of toxicity that dominate in the ecotoxicological literature. On the basis of the data generated here, it appears that such toxicity data at constant temperatures will provide a good assessment of effects under variable conditions in those cases where the range of the environmental variable does not include conditions beyond the physiological limits of tolerance of the species. Hence, rather than being innately stressful in its own right, temperature fluctuation is merely a vehicle that may on occasion result in exposure to a stressful condition. When temperatures remain constant and in the normal physiological range, the impacts of Cu toxicity appear to be marginal, with toxicity only affected as temperature approach or exceed physiological limits. Fluctuations, even when they may result in some stress exposure themselves, do not necessarily result in sensitisation. Indeed, as seen here a version of a “stress hardening” response may also occur [64,65]. Hence, consideration of fluctuating temperature does not in itself need a rethink of our approach to chemical risk assessment as a previously uncharacterised source of uncertainty.

## Supporting Information

S1 Fig The DEBkiss model structure. A schematic diagram for the energy flows in DEBkiss. The equations behind the fluxes are given in Table 1.
(TIF)

S2 Fig The effects of Cu exposure on traits responses in *C*. *elegans* in treatments with an average temperature of 12°C. The five different endpoints: Final brood size (A), final body length (A), time to first egg (B), lifespan (D) and Population Growth Rate (PGR) (E) for the constant 12°C treatment (filled symbols) and the 12 ± 4°C (grey symbols) as a function of Cu concentrations in the agar. Data are given as mean ± s.e.m. and are described with a three parameter log-logistic concentration response model, except for TFE. The parameters are given in Table 3, together with the concentration-response parameters of the other temperature treatments.
(TIF)

S3 Fig The effects of Cu exposure on traits responses in *C*. *elegans* in treatments with an average temperature of 10°C. The five different endpoints: Final brood size (A), final body length (A), time to first egg (B), lifespan (D) and Population Growth Rate (PGR) (E) for the constant 20°C treatment (filled symbols) and the 20 ± 4°C (grey symbols) as a function of Cu concentrations in the agar. Data are given as mean ± s.e.m. and are described with a three parameter log-logistic concentration response model, except for TFE. The parameters are given in Table 3, together with the concentration-response parameters of the other temperature treatments.
(TIF)
