# Supplementary figures and images for: Variable Temperature Stress in the Nematode Caenorhabditis elegans (Maupas) and Its Implications for Sensitivity to an Additional Chemical Stressor
Source: PLoS One. 2016 Jan 19;11(1):e0140277. doi: 10.1371/journal.pone.0140277 (PMC4718611; doi:10.1371/journal.pone.0140277)

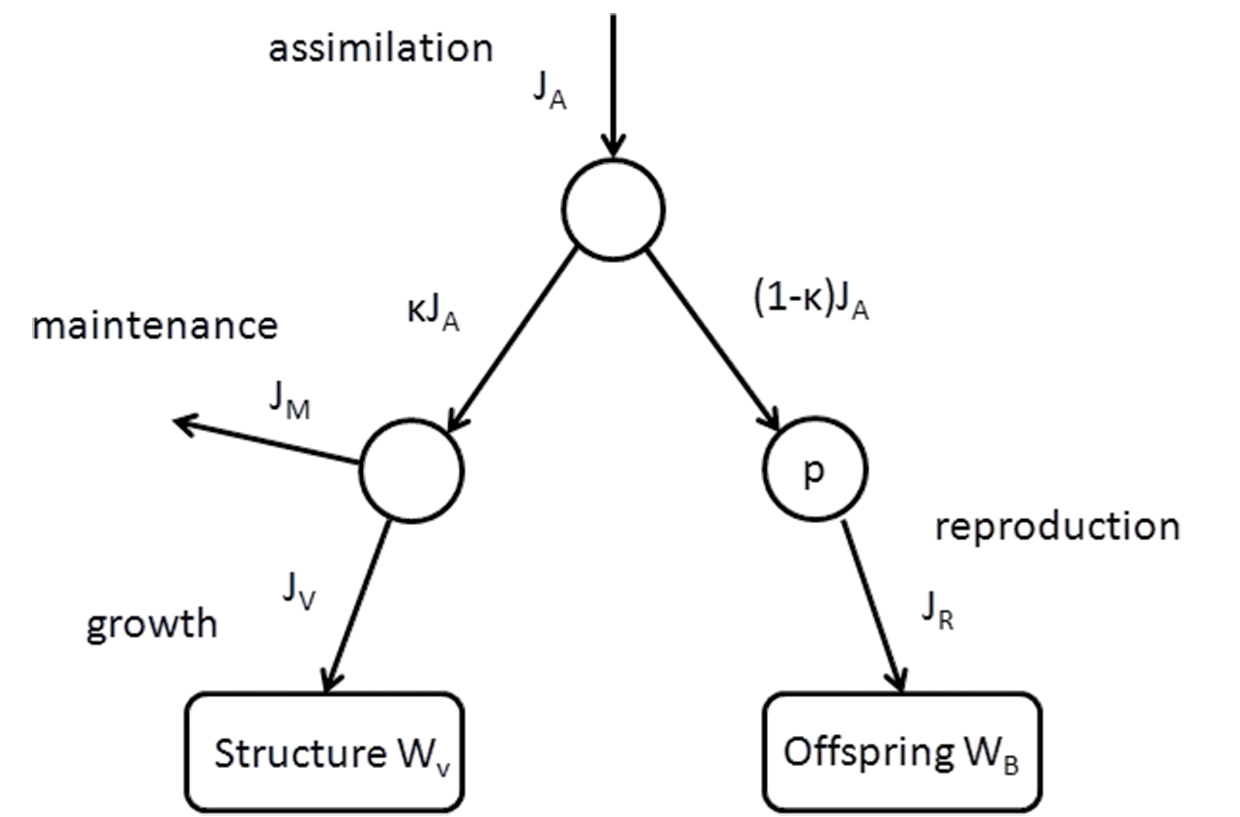

Supplement: S1 Fig — A schematic diagram for the energy flows in DEBkiss. The equations behind the fluxes are given in Table 1. (TIF) [file pone.0140277.s001.tif]

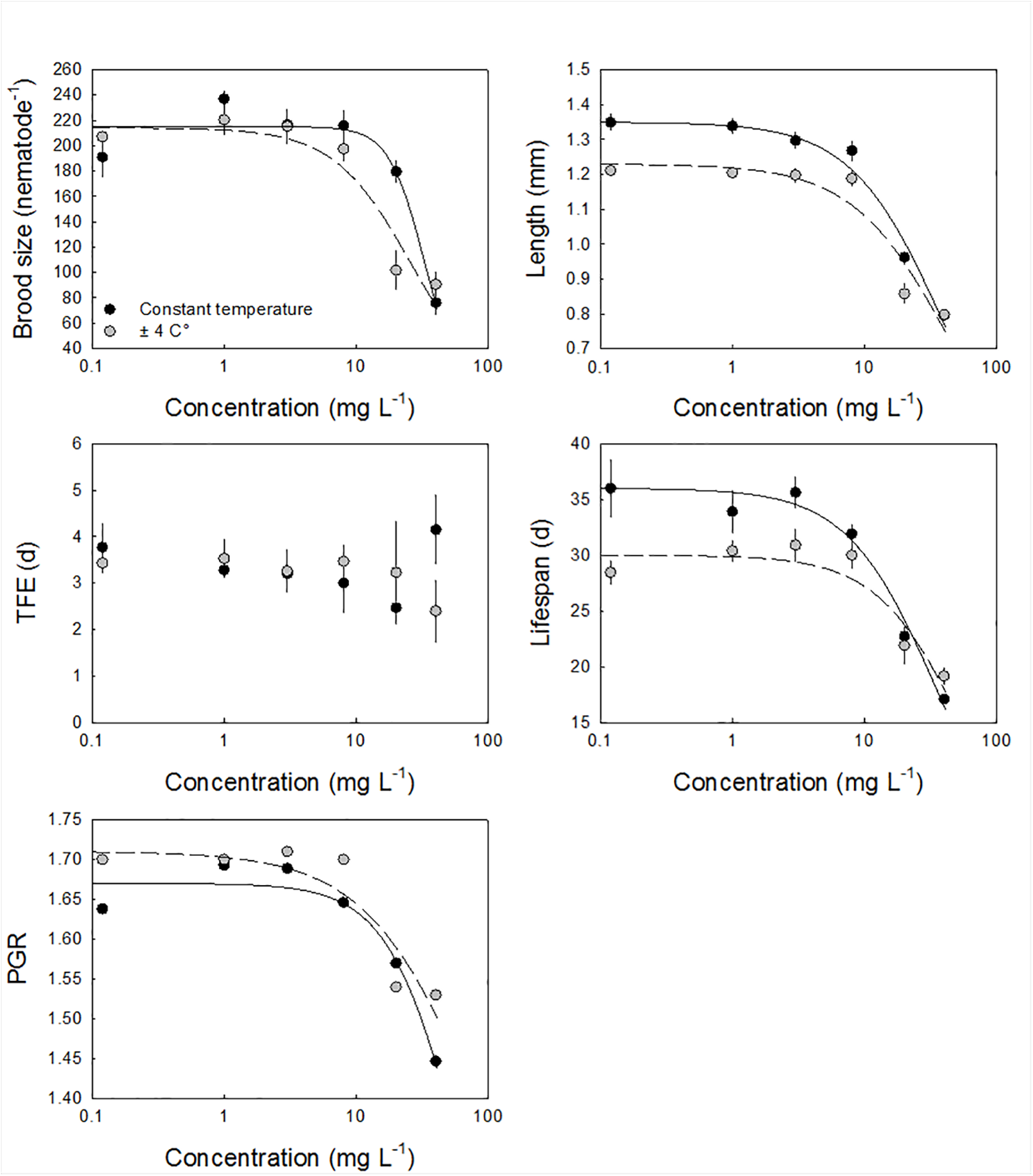

Supplement: S2 Fig — The five different endpoints: Final brood size (A), final body length (A), time to first egg (B), lifespan (D) and Population Growth Rate (PGR) (E) for the constant 12°C treatment (filled symbols) and the 12 ± 4°C (grey symbols) as a function of Cu concentrations in the agar. Data are given as mean ± s.e.m. and are described with a three parameter log-logistic concentration response model, except for TFE. The parameters are given in Table 3, together with the concentration-response parameters of the other temperature treatments. (TIF) [file pone.0140277.s002.tif]

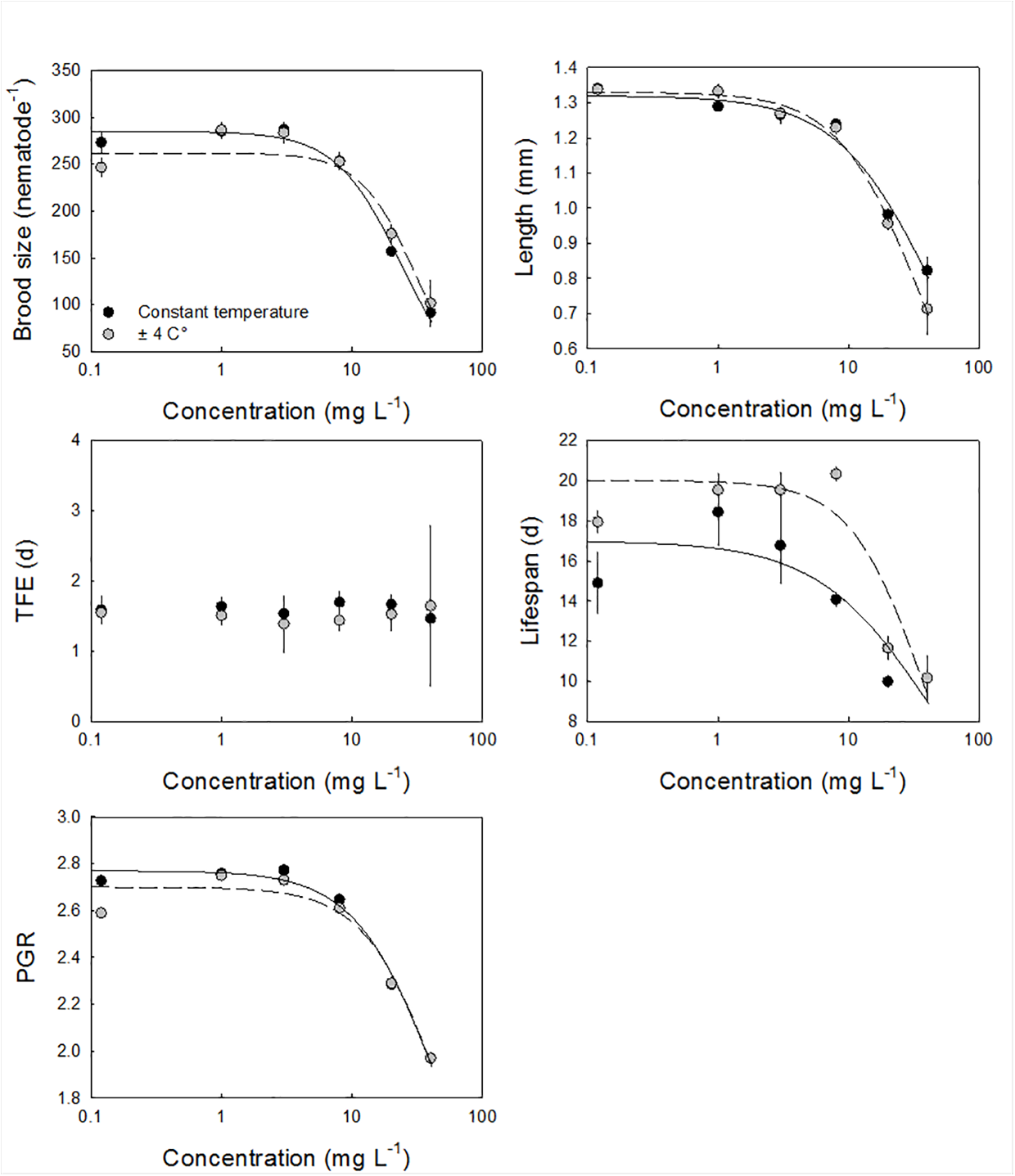

Supplement: S3 Fig — The five different endpoints: Final brood size (A), final body length (A), time to first egg (B), lifespan (D) and Population Growth Rate (PGR) (E) for the constant 20°C treatment (filled symbols) and the 20 ± 4°C (grey symbols) as a function of Cu concentrations in the agar. Data are given as mean ± s.e.m. and are described with a three parameter log-logistic concentration response model, except for TFE. The parameters are given in Table 3, together with the concentration-response parameters of the other temperature treatments. (TIF) [file pone.0140277.s003.tif]
